# Myocardial Bridging: Two Different Clinical Presentations in Young Males Involving Left Anterior Descending Coronary Artery

**DOI:** 10.7759/cureus.26134

**Published:** 2022-06-20

**Authors:** Samaj Adhikari, Arjun Mainali, Binit Aryal, Puspa B Bista, Saujan Devkota, Nicole Gousy, Tutul Chowdhury, Alix Dufresne

**Affiliations:** 1 Internal Medicine, Interfaith Medical Center, New York City, USA; 2 Medicine, Interfaith Medical Center, New York City, USA; 3 Medicine, American University of Antigua, New York City, USA; 4 Cardiology, Interfaith Medical Center, New York City, USA

**Keywords:** stable angina, rare variant, coronary artery variants, left anterior descending artery, myocardial bridging

## Abstract

Myocardial bridging is a rare anatomical variant that can lead to detrimental cardiac consequences when undiagnosed and untreated. This rare variant can induce anginal-type symptoms due to disrupted blood flow to the myocardium during systole. The patients presented in this report of two cases had previously undiagnosed myocardial bridging of the left anterior descending artery, however clinically, they presented quite differently. Here we present two cases discussing the course of diagnosis and treatment of myocardial bridging of these two patients. The goal of this case report is to highlight the significant cardiovascular injuries that can be a result of undiagnosed myocardial bridging.

## Introduction

Myocardial bridging is a rare anatomical variant in the coronary artery potentially leading to myocardial ischemia, left ventricular dysfunction, cardiac arrhythmia, and even sudden cardiac deaths [1-4]. Surprisingly, a higher prevalence is revealed in autopsy compared to coronary angiogram [5]. Similarly, a higher prevalence is noted in cardiac transplant recipients and patients with hypertrophic obstructive cardiomyopathy (HOCM) [6,7].

Patients become symptomatic when the coronary vessel tunneling through the myocardium gets compressed during systole. Patients are medically managed with beta-blockers and calcium-channel blockers and further managed with surgical interventions with myotomy, and/or coronary artery bypass grafting (CABG) and stenting of tunneled arteries [8,9]. We report two cases of obese patients who presented with chest pain. Further investigation later revealed myocardial bridging in coronary angiograms.

## Case presentation

A 40-year-old male with a past medical history of hypertension and obesity came to the emergency department (ED) with complaints of progressively worsening shortness of breath and intermittent exertional central chest pain for the past six months. These symptoms had progressively worsened over the last few days resulting in severe impairment of his functional capacity and routine daily activities. These symptoms were additionally associated with swelling of the legs bilaterally, orthopnea, paroxysmal nocturnal dyspnea, and occasional nonproductive cough. He denied palpitation and any episodes of syncope. Review of systems was otherwise negative. He has a smoking history of 8 pack-years, drinks socially, and occasionally smokes marijuana. Family history was significant for heart disease in the mother and brother; however, the nature of the disease was unknown but he denied any sudden cardiac death in the family.

**Figure 1:**
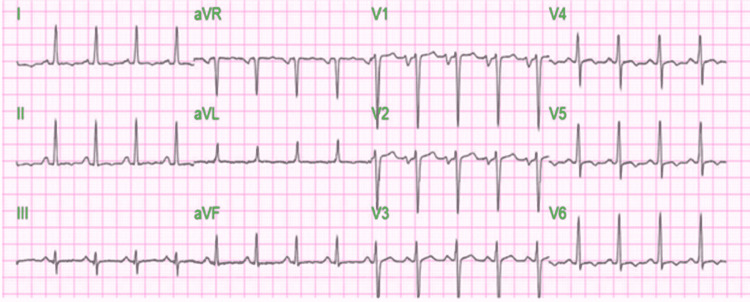
EKG taken during the time of admission with heart rate: 106, QTc: 454 with no significant ST/T wave changes

On physical examination, he was obese with a BMI of 36.5. Triage vitals showed blood pressure of 138/97 mmHg, heart rate (HR) of 114 beats/min, respiratory rate of 23, and oxygen saturation of 97% on room air. Jugular venous pressure was elevated. A cardiovascular exam revealed sinus tachycardia without any murmur, rub, or gallop. Bilateral vesicular breath sounds were heard with mild wheezing and bilateral basal crepitation. He had bilateral pitting edema. On admission, brain natriuretic peptide (BNP) was 1865 pg/mL, high-sensitivity troponin was 49.5 ng/L, D-dimer 709, high-density lipoprotein (HDL) was 22 mg/dL, low-density lipoprotein (LDL) was 115 mg/dL, triglycerides (TG) were 89 mg/dL, and total cholesterol was 155 mg/dL. Urine toxicology was positive for cannabinoids. All other lab parameters, including complete blood count (CBC), comprehensive metabolic panel (CMP), and thyroid-stimulating hormone (TSH), were within normal limits. EKG showed: sinus tachycardia, QTc 454 as described in Figure 1.

**Figure 2:**
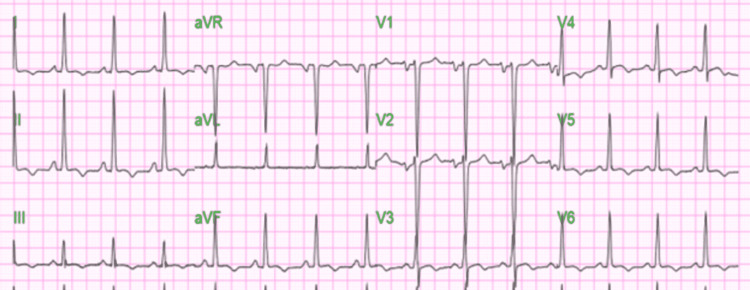
EKG showing heart rate: 88, QTc: 471 with new T wave inversion in leads II, III, and aVF

A chest x-ray showed borderline vasculature with cardiomegaly. CT pulmonary angiogram (CTPA) was negative for pulmonary embolism and aortic dissection. Echocardiogram showed severely reduced systolic function ejection fraction (EF): 16%, diffuse hypokinesis, decreased left ventricular diastolic compliance, increased left atrial pressure, and a mild mitral and tricuspid regurgitation. Furosemide 40 mg every 12 hours, lisinopril 20 mg daily, aspirin 81 mg daily, and atorvastatin 20 mg daily were subsequently started and he was admitted to telemetry for continuous cardiac monitoring. A beta-blocker was not started initially in view of acute exacerbation of heart failure. Troponin and EKG were trended and were not suggestive of acute ischemic changes at the time of admission. On day 2 of admission, he complained of constant severe central chest pain. High-sensitivity troponin was 681.2 ng/L, and EKG showed new T wave inversion in leads II, III, and aVF as described in Figure 2.

**Figure 3:**
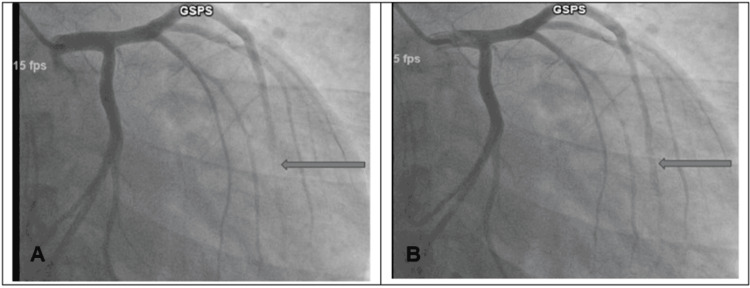
Left coronary angiogram showing severe myocardial bridging in the distal left anterior descending artery as shown by the arrow (A) during systole and with normal flow in diastole as shown by the arrow (B)

He was given a loading dose of aspirin 325 mg, nitrates (nitroglycerine 0.4 mg sublingual), morphine 2 mg, and a therapeutic dose of Lovenox 120 mg and was transferred for urgent cardiac catheterization. Cardiac catheterization revealed severely depressed global left ventricular function without significant coronary artery disease (CAD). Severe myocardial bridging was present in the distal left anterior descending artery (LAD), as shown in Figure 3.

Medications were subsequently optimized to metoprolol-XL 50 mg daily, spironolactone 25 mg daily, Entresto 24-26 two times daily, dapagliflozin 10 mg daily, aspirin 81 mg daily, and atorvastatin 20 mg daily. He was discharged with a life vest and was instructed to follow up with the cardiology clinic for a device check. On follow-up, he improved clinically with an increase in his functional capacity. The follow-up device check did not reveal any significant arrhythmia.

Case 2

A 25-year-old male with a past medical history of uncontrolled type 2 diabetes, hypertension, childhood bronchial asthma, dyslipidemia, and obesity presented to the ED with complaints of acute pressure-like central chest pain for 15-20 minutes with an onset of 1 hour before the presentation. This was associated with diaphoresis and one episode of non-projectile, non-bilious vomiting. He denied shortness of breath, cough, palpitation, and syncopal episode. All other reviews of systems were unremarkable. He has a smoking history of 10-15 cigarettes/day for 3-4 years with a pack-year of two. He is a social drinker and takes marijuana occasionally. Family history is not significant for any cardiac disease or sudden cardiac death.

**Figure 4:**
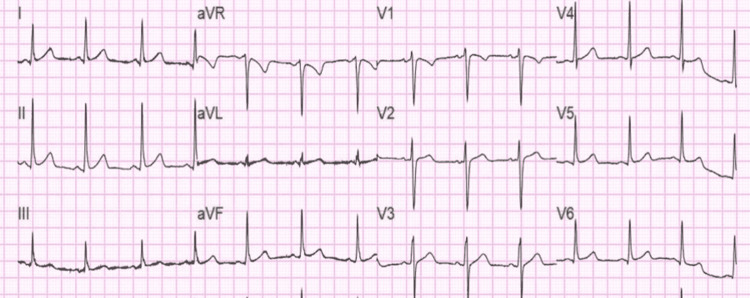
EKG taken during the time of admission showing heart rate: 79, QTc: 438 with no significant ST/T wave changes

On physical examination, he was obese with BMI of 38. Triage vitals included: blood pressure of 148/104 mmHg, HR of 70 beats/min, respiratory rate of 16, and oxygen saturation of 98% on room air. On cardiovascular exam: normal S1/S2, with a regular rate and rhythm with no murmurs, rubs, or gallops were observed. Chest exam: bilateral vesicular breath sounds with no added sounds. All other physical findings were normal. Admission labs were significant for elevated high-sensitivity troponin of 1833 ng/L, D-dimer of 587, random blood glucose of 254 mg/dL, HDL 26 mg/dL, LDL 185 mg/dL, TG: 276 mg/dL, total cholesterol 266 mg/dL, and urine toxicology were positive for cannabinoids. All other labs, including CBC, CMP, BNP, and lipase, were within normal limits. EKG showed HR of 79, QTc: 438 but no ST-T wave changes as in Figure 4.

**Figure 5:**
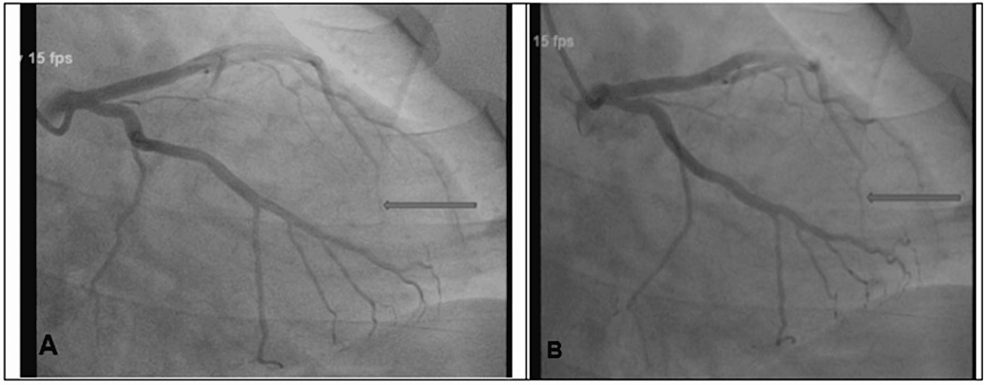
Left coronary angiogram showing myocardial bridging in the mid left anterior descending artery as shown by the arrow (A) during systole and with normal flow as shown by the arrow (B)

The patient received a loading dose of aspirin 325 mg, clopidogrel 225 mg, atorvastatin 40 mg, and one therapeutic dose of Lovenox 100 mg. A bedside echo was done, which was normal with EF: 55-60%. CTPA was negative for pulmonary embolism and aortic dissection. High-sensitivity troponin trended up to 2749 ng/L, however, repeat EKG did not show any new changes. He was immediately transferred for cardiac catheterization. Cardiac catheterization was performed and revealed no significant CAD with normal left ventricular ejection fraction (LVEF): 55% but revealed bridging of the mid LAD segment as described in Figure 5.

He was discharged with oral diabetic metformin 500 mg every 12 hours, lisinopril 10 mg daily (for hypertension), and atorvastatin 10 mg daily for diabetes mellitus. On the second day of admission, approximately 8-9 hours post-cardiac catheterization, he left against medical advice even after extensive counseling.

## Discussion

Myocardial bridging commonly involves the LAD artery as in our cases, though rarely the right coronary artery is involved. [10] Patients with myocardial bridge typically present with angina symptoms. Compression of the tunneled vessel during systole with delayed diastolic reopening produces angina equivalent symptoms consistent with the presentation of our patients [11]. Our first patient, however, presented with progressive shortness of breath with echocardiogram showing severely reduced systolic function, diffuse hypokinesis, and decreased left ventricular diastolic compliance. Cai et al. reported a higher prevalence of left ventricular systolic dyssynchrony (LVSD) among patients with myocardial bridging [12]. Myocardial stunning with akinesis of the apex, anterolateral, and inferoapical wall was previously reported in a patient with myocardial bridging [13].

In an earlier study, myocardial fibrosis and interstitial edema were significantly associated among patients with hemodynamically significant myocardial bridges in sudden cardiac death [14]. Myocardial remodeling secondary to repeated ischemia due to severe distal LAD myocardial bridging could have possibly contributed to the severe decline in systolic function in our first case. There was no significant family history of cardiac disease, an echocardiogram was nonsuggestive of hypertrophic cardiomyopathy, thyroid function test was within normal limit, and no signs and symptoms of a recent viral illness or systemic manifestations of infiltrative, storage, or autoimmune diseases to suggest nonischemic cardiomyopathy. Cardiac MRI could have been done to rule out other rare causes of nonischemic cardiomyopathy, which seems very unlikely.

Patients with myocardial bridging presenting with angina symptoms can have normal resting EKG like in our second case. Even on exercise stress tests, the absence of EKG changes is common in myocardial bridging, however, patients can present with angina [15]. Akdemir et al. reported acute anteroseptal myocardial infarction with ST elevation on EKG in a patient with myocardial bridging [16].

The degree of vessel narrowing, depth, and number of tunneled arteries determine the manifestations and subsequent complications of the myocardial bridging [17,18].

Coronary angiography is the gold standard for diagnosing myocardial bridging. In coronary angiography, the systolic compression of the tunneled artery is classically depicted as a “milking effect”, with retrograde blood flow on systole and subsequent antegrade flow during diastole. Intravascular US and intracoronary Doppler sonogram are other diagnostic modalities of choice. Our patients underwent coronary angiography which showed myocardial bridging without significant CAD.

Recent advances in cardiac imaging with cardiac CT, cardiac MRI, can also be used in making the diagnosis [19,20]. Cardiac CT is an alternative for patients with a low pretest probability for coronary atherosclerosis.

Management of myocardial bridging involves reducing intramyocardial artery compression by shortening the systolic phase and lengthening the diastolic phase, which can be achieved with the administration of beta-blockers and calcium-channel blockers. Sublingually administered nitroglycerine could even exacerbate the angina symptoms as reported in previous studies [21,22]. Our patient, however, did not have a significant worsening of angina symptoms after the administration of nitrate. Antiplatelet therapy can be administered as a supplement if atherosclerosis is present. Intracoronary stenting, surgical myotomy, and/or CABG may be considered if symptoms continue despite medical management. Arguably, CABG is generally recommended when an endovascular failure occurs and also in deeper and extensive myocardial bridges [23]. Cases of stent fracture following stenting of the myocardial bridge have been reported earlier [24]. Although both of our patients had significant cardiovascular risk factors, obstructive CAD was not detected on the angiogram so revascularization was not considered. Long-term follow-up is required to closely monitor the patients and intervene timely.

Other common differentials including MINOCA (myocardial infarction with nonobstructive coronary arteries), hypercoagulability, vascular spasm, or coronary dissection should be considered when evaluating the cause of chest pain syndromes in young patients with low pretest probability for atherosclerosis. Although coronary angiograms were negative for obstructive CAD in both of our cases, full imaging studies should be considered to diagnose coronary anomalies in patients with a low pretest probability of atherosclerosis who come with angina equivalents, as these defects can have serious consequences. Our cases of two young patients are unique with similar cardiovascular risk factor profiles and with the overlapping presentation of angina symptoms but yet different left ventricular systolic function leading to severe clinical heart failure as demonstrated in our first patient.

## Conclusions

Myocardial bridging is one of the infrequent benign congenital anomalies featuring coronary arteries where an epicardial coronary artery follows an intramyocardial course, commonly involving LAD. Changes in flow dynamics from this condition can result in accelerated atherosclerosis in the portion proximal to the bridged segment sparing the bridging segment itself. Though several detrimental cardiac conditions including sudden cardiac death have been considered as a consequence of myocardial bridging, making it attributable as the only cause of the events is still debatable. Cardiac computed tomography angiography, intravascular ultrasound, and CT-based noninvasive fractional flow reserve could be proved helpful in clinical practice in terms of the study of this clinical attribute but yet need more evidence. Medical therapy is preferred as first-line therapy whereas interventional procedures have also been attempted for refractory symptoms. Further correlation and research should be encountered to define prevalent patient communities to provide most benefit from intervention as well as to categorize best treatment strategy.
